# Temporal and spatial dynamics of Bacteria, Archaea and protists in equatorial coastal waters

**DOI:** 10.1038/s41598-019-52648-x

**Published:** 2019-11-08

**Authors:** Caroline Chénard, Winona Wijaya, Daniel Vaulot, Adriana Lopes dos Santos, Patrick Martin, Avneet Kaur, Federico M. Lauro

**Affiliations:** 1 Asian School of the Environment, Nanyang Technological University, 50 Nanyang Avenue, Singapore, 639798 Singapore; 2 Sorbonne Université, CNRS, UMR7144, Ecology of Marine Plankton team, Station Biologique de Roscoff, 29680 Roscoff, France; 3 GEMA Center for Genomics, Ecology & Environment, Universidad Mayor, Camino La Pirámide, 5750 Huechuraba, Santiago Chile; 4 Singapore Centre for Environmental Life Sciences Engineering (SCELSE), Nanyang Technological University, 60 Nanyang Dr, Singapore, 637551 Singapore

**Keywords:** Molecular ecology, Water microbiology, Microbial ecology

## Abstract

Singapore, an equatorial island in South East Asia, is influenced by a bi-annual reversal of wind directions which defines two monsoon seasons. We characterized the dynamics of the microbial communities of Singapore coastal waters by collecting monthly samples between February 2017 and July 2018 at four sites located across two straits with different trophic status, and sequencing the V6-V8 region of the small sub-unit ribosomal RNA gene (rRNA gene) of Bacteria, Archaea, and Eukaryota. Johor Strait, which is subjected to wider environmental fluctuations from anthropogenic activities, presented a higher abundance of copiotrophic microbes, including Cellvibrionales and Rhodobacterales. The mesotrophic Singapore Strait, where the seasonal variability is caused by changes in the oceanographic conditions, harboured a higher proportion of typically marine microbe groups such as Synechococcales, Nitrosupumilales, SAR11, SAR86, Marine Group II Archaea and Radiolaria. In addition, we observed seasonal variability of the microbial communities in the Singapore Strait, which was possibly influenced by the alternating monsoon regime, while no seasonal pattern was detected in the Johor Strait.

## Introduction

Marine planktonic communities harbour representatives from all three domains of life (Bacteria, Archaea and Eukaryota). Together, these organisms perform a range of global biological and geochemical processes^1^. In contrast to the open ocean, coastal environments are influenced by local disturbances such as freshwater land run-off, land to sea transfer of nutrients and organic matter through river discharge and mixing in shallow areas induced by tidal currents, in addition to seasonal cycles^2,3^. These variable conditions may lead to complex dynamics where patterns of reoccurring microbial communities are harder to access and to predict.

In temperate waters, annual cycles of plankton communities have been studied for a long time^4,5^. The seasonal changes in microbial community composition and biomass are often attributed to responses to changes in environmental conditions driven by seasonal climate cycles^6–9^. For example, Lambert *et al*.^7^ demonstrated a strong rhythmicity in Bacteria, Archaea and protist communities despite sporadic meteorological events and irregular nutrient availability at a coastal site situated in the North Western Mediterranean Sea. In contrast, few studies have investigated the seasonal dynamics of coastal microbial communities in equatorial waters.

Equatorial waters are subjected to monsoons, periods when the prevailing winds over land and adjacent ocean areas reverse directions on a seasonal basis in response to differences in heating patterns between land and ocean. These differences alter the patterns of rising and sinking air near the equator, resulting in the seasonal migration of the intertropical convergence zone (ITCZ), an area of low atmospheric pressure where the Northeast and Southeast Trade Winds meet^10^. By influencing vertical mixing^11^, upwelling^12^ and advective transport^13^, monsoon systems have previously been shown to influence phytoplankton dynamics in tropical waters^11,14,15^. Miki *et al*.^11^ demonstrated a higher concentration of chlorophyll during the NE monsoon than the SW monsoon in the Sulu Sea off the southwest side of the Philippines. A similar trend in chlorophyll concentration was also observed in the Strait of Malacca^14^.

In Singapore, a highly urbanized island city state of South East Asia located just one degree North of the equator, a bi-annual reversal of wind directions and two monsoon seasons named after the prevailing wind direction^10^ characterize the annual meteorological conditions in the island. The Northeast Monsoon (NE Monsoon), generally occurring from December to early March, brings primarily north-easterly winds and long periods of heavy rain across the region, while the Southwest Monsoon (SW Monsoon), from June to September, exhibits south-westerly winds with higher temperatures, scattered showers and thunderstorms mostly occurring in the afternoon. Inter-monsoon periods are generally less windy and experience lower precipitation. Singapore is also surrounded by two main bodies of water with different trophic status: the Singapore Strait in the South and the Johor Strait in the North. The Singapore Strait exhibits oceanic conditions and is influenced by strong tidal currents up to 2 m s^−1^ and alternate oceanic influxes from the South China Sea and the Java Sea^16^. The Johor Strait, less than 1 km wide, is subjected to greater environmental fluctuations resulting from a combination of anthropogenic activities, sporadic riverine inputs and reduced tidal mixing^17^. Recurrent spring/summer blooms have been reported during the inter-monsoon months April/May in the years of 1935, 1948, 1949 and 1968^18^. More recent studies either applied morphological identification methods to a restricted number of taxa^17^ or used techniques such as flow cytometry and pigments analysis that have limited taxonomic resolution^19,20^ to investigate the dynamics of the plankton communities in Singapore coastal waters.

Here, we present an 18-month study that uses SSU rRNA gene metabarcoding to characterize the temporal variation of the marine microbial community in Singapore waters. We observed taxonomic compositions reflecting differences in trophic status between the two Straits and identified a seasonal signature in the microbial community composition of the Singapore Strait, probably linked to the monsoonal current reversal. In contrast, no seasonal patterns, but large month-to-month changes, in beta-diversity were observed in the Johor Strait, suggesting that localized pulse disturbances might be responsible for shaping community composition.

## Materials and Methods

### Sampling sites and water collection

Surface water (~1 m depth) was collected with a submersible pump monthly from February 2017 to July 2018 at four different stations around Singapore (Fig. S1, Supplementary Data S1). Sampling was performed within two days of neap tide, except for samples #21 collected during the full moon (28 June 2018). Two stations were in the Singapore Strait, St. John (1.2383°N, 103.8536°E) and East Coast (1.2972°N, 103.9207°E), and two in the Johor Strait, Pasir Ris (1.3886°N, 103.9515°E) and Sembawang (1.4716°N, 103.8051°E). Four monsoon periods were identified using the prevailing wind direction from the annual climatological report for 2017 and 2018 from the Meteorological Service of Singapore^21^: Northeast (NE), inter-monsoon 1 (IM-1), Southwest (SW), and inter-monsoon 2 (IM-2). A month was assigned to the NE or SW Monsoon if the wind recorded at Changi airport blew for more than 50% of the month from either the first quadrant (NE) or third quadrant (SW) direction, respectively, and assigned to the IM periods in all other cases.

Temperature and salinity were measured at ~1 m using a Eureka Water Probes Manta + Trimeter Multiprobe. Chlorophyll was measured using an AquaFlash Handheld Active Fluorometer (Turner Designs). For dissolved inorganic nutrients, water was syringe-filtered (0.22 *μ*m Acrodisc, PALL) into acid-washed, polypropylene centrifuge tubes, immediately flash-frozen in liquid nitrogen, and stored at −20 °C until analysis. For microbial community samples, 1L of seawater was first filtered through a 150 *μ*m net mesh filter to remove large detritus and zooplankton, and then the biomass was collected on 0.22 *μ*m Sterivex filters (Millipore). The Sterivex filters were immediately stored at −80 °C until further analysis. Samples for flow cytometry (980 *μ*L) were fixed for 15 min at 4 °C in the dark with 25% electron microscopy (EM) grade glutaraldehyde (0.5% final concentration; Sigma-Aldrich), flash-frozen in liquid nitrogen and stored at −80 °C until analysis.

### Nutrient analysis

Samples for dissolved inorganic macronutrients, NO_3_ + NO_2_ (referred to as NO_x_ hereafter), NO_2_, NH_4_, PO_4_, and Si(OH)_4_ were thawed at room temperature and immediately measured on a SEAL AA3 segmented-flow autoanalyser according to SEAL methods for seawater analysis. NH_4_ was measured fluorometrically^22^. For samples collected on 3 April 2017, 02 May 2017 and 31 May 2017, the dissolved inorganic macronutrients were measured using the APHA^23^ method and Si (4500-SiO_2_ Flow injection analysis for Molybdate-Reactive Silicate) as a service provided by DHI-Singapore Seawater. Total dissolved inorganic nitrogen (DIN) was calculated as NO_x_ + NH_4_.

### Enumeration of prokaryotes

Prokaryotes were enumerated in duplicate water samples by flow cytometry using a CytoFLEX (Beckman Coulter, Singapore) equipped with blue (488 nm) and violet (405 nm) lasers. Prior to analysis, samples were thawed and diluted (between 5–10x) with 0.2 *μ*m filtered sterile 10 mM Tris, 1 mM ethylene-diamine-tetra-acetic acid buffer (pH 8.0) and stained with SYBR Gold (Life Technologies, Singapore). Samples were incubated in the dark for 15 minutes prior to analysis. The analysis was performed for 1 minute at medium flow rate (~30 *μ*L min^−1^) with an event rate of ~200–1200 particles per second with a threshold set on the green fluorescence channel (525/40 BP). Prokaryotes were discriminated based on their signature on a scatter plot of green fluorescence against violet side-scatter using the CytExpert software provided with the CytoFLEX.

### DNA extraction and 16S/18S PCR amplification and sequencing

DNA was extracted from the Sterivex filters using a modified protocol for MoBio PowerSoil kit (MoBio Laboratories, Carlsbad, CA), as previously published by Jacobs *et al*.^24^. Briefly, the Sterivex filter casing was broken open and the filter was cut with a sterile razor blade into small lengthwise strips and placed into the PowerBead tube. 60 *μ*L of the manufacturer solution C1 were added to the tube which was incubated for 10 min at 70 °C with constant shaking (500 rpm) twice and vortexed at maximum speed between each incubation. Following the last incubation, 700 *μ*L of phenol-chloroform-isoamyl alcohol was added into the PowerBead tube and vortexed at maximum speed for 10 min. The PowerBead tube was then centrifuged at 10,000 g for 30 seconds and 800 *μ*L of supernatant transferred to a new collection tube. The remaining steps for the DNA extraction followed the manufacturer’s instructions. DNA was eluted into 100 *μ*L of nuclease-free water and quantified using a Qubit 2.0 fluorometer with the dsDNA Broad Range assay kit (Invitrogen, Singapore). Amplicon libraries were then generated using a modified version of the Illumina 16S Metagenomic Sequencing Library Preparation Protocol^25^. Universal primer pairs (926wF: 5′-AAACTYAAAKGAATTGRCGG-3′ and 1392R: 5′-ACGGGCGGTGTGTRC-3′) targeting the V6-V8 hyper-variable region of the 16S/18S ribosomal RNA gene with an Illumina-specific overhang were used^26^. These primers have been tested previously in silico against the Silva database and shown to amplify 84%, 71% and 91% of rRNA gene sequences from Bacteria, Archaea and Eukaryota, respectively^27^. For each sample, triplicate PCR reactions obtained with 22 cycles were pooled and purified using Agencourt AMpure XP beads (Beckman Coulter, Singapore). Products from this first step were then sent to the sequencing facility at the Singapore Centre for Life Science and Engineering (SCELSE) where a second round of PCR was performed to add dual barcodes to each amplicon library. Afterwards, PCR products were purified with Agencourt AMpure XP beads, pooled at equal volume and sequenced on an Illumina MiSeq machine with MiSeq Reagent Kit v3 chemistry (2 × 300 bp).

### Sequence analysis

The raw sequencing data was initially processed by removing primers with cutadapt^28^. Paired-end joining, denoising and taxonomic assignment of Amplicon Sequence Variants (ASV) were performed using Quantitative Insights Into Microbial Ecology (QIIME) release 2018.6^29^. Briefly, after importing the sequence data into the QIIME2 environment, the denoising and pair-end joining were performed using DADA2^30^. After discarding all the ASVs with less than 10 sequence representatives across the 70 samples, phylogenetic relationships were inferred by aligning representative sequences with MAFFT^31^, filtering the alignment and constructing phylogenetic trees using fasttree^32^ with a midpoint root. Taxonomic classification was performed using the classify-sklearn method^33^ using the SILVA version 132 database as a reference. All ASVs identified as eukaryotic (nuclear, plastid) were further assigned against the PR^2^ database^34^ version 4.12.0 (https://github.com/pr2database/pr2database) using the RDP naive Bayesian classifier^12^ as implemented in the R *dada2* package^30^. ASVs with a bootstrap value lower than 90% at the supergroup level were discarded. For statistical analyses, Metazoa (mostly copepods) were removed from the final ASV table because they are likely to represent eggs or organismal fragments that were not captured by the pre-filtering step. Similarly, eukaryotic plastid sequences (189 ASVs) were not considered to avoid counting photosynthetic organisms twice. The final ASV table contained a total of 2,571 ASVs (For more details see https://github.com/slimelab/Singapore-metabarcodes).

### Statistical analyses

The Shannon index (H′) and richness were calculated with the software R^35^ using the *vegan* package^36^. Pairwise community dissimilarity was calculated using Bray-Curtis distance as implemented in the *vegan* package^36^. The vegan package was also used to generate non-metric multidimensional scaling (nMDS) plots using the metaMDS function and pairwise comparisons between the different locations (Johor Strait, Singapore Strait) and monsoon periods using the ANOSIM function. An Envfit analysis (also available in the vegan package) were generated using the following parameters: salinity, temperature, chlorophyll, prokaryote abundance, PO_4_, DIN, Si(OH)_4_, NH_4_ and amount of rainfall seven days (rain-7) prior to the sampling day. The differential abundance of relevant ASVs was extrapolated using Gneiss^37^, with Ward’s correlation clustering as a function of monsoon period for the Singapore Strait. The ASVs contributing significantly to the difference between the two monsoon regimes were computed using the DESeq2 package^38^ using a threshold for the adjusted (following Benjamini and Hochberg, 1995^39^) p-value of 0.01. The magnitude of changes occurring in the community during the time series was inferred with the first-distances method^40^ applied to the unweighted Unifrac distance matrix^41^. Compared to other distance metrics, Unifrac computes the magnitude of the differences in the phylogenetic trees between pairs of samples. When coupled with the first-distances approach using time series data^40^, it is a measure of how many taxa have disappeared and how many new taxa have appeared from one month to the next. The results of this analysis were graphically displayed on a volatility plot.

## Results

### Physico-chemical parameters in Singapore coastal waters

**Figure 1:**
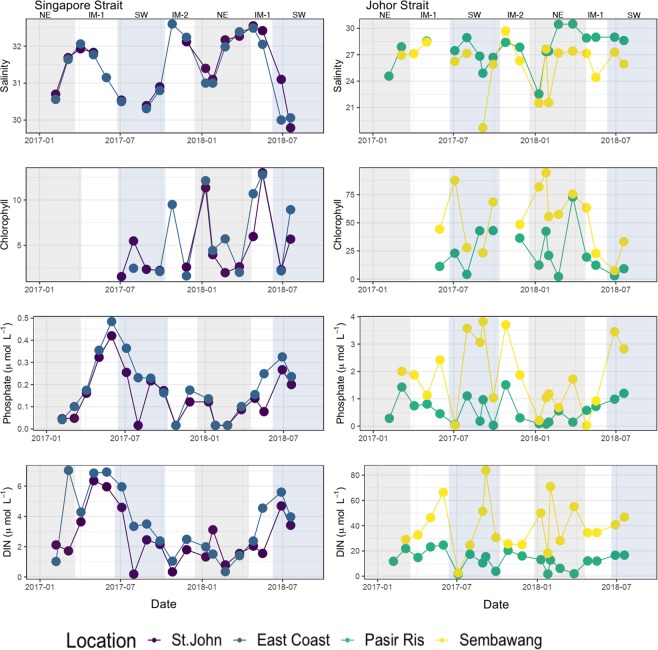
Salinity, chlorophyll, phosphates and DIN (dissolved inorganic nitrogen) during the 18-month time series in Singapore coastal waters. Highlights in grey and blue represent NE and SW monsoon, respectively.

Despite their proximity and physical connection, the Singapore and Johor Straits exhibit different environmental conditions (Fig. 1, Fig. S2, Supplementary Data S1). Temperature showed little variation, but was slightly higher in the Johor (29.0–32.8 °C) than the Singapore (27.1–30.6 °C) Strait (Fig. S2A). The lowest temperatures were consistently found during the NE monsoon. Much larger variability was observed in salinity: the two sites in the Singapore Strait were very similar to each other, with salinity ranging from 29.8 to 32.6 and lowest values during the SW monsoon (Fig. 1). In contrast, salinity was lower and more variable in the Johor Strait, ranging from 18.1 to 30.3, with lowest values mostly during the NE monsoon. Sembawang, the sampling site closest to the causeway between Singapore and Malaysia, typically had lower salinity than Pasir Ris located more to the East (Fig. 1).

Dissolved nutrient concentrations also differed strongly between the Singapore and Johor Straits: NO_x_, NO_2_, NH_4_ and PO_4_ were typically 2–10-fold higher in the Johor compared to the Singapore Strait, while Si(OH)_4_ was on average more similar, but with higher variability in the Johor Strait (Fig. 1, Supplementary Data S1, Fig. S2). The Singapore Strait showed more oligo- to mesotrophic characteristics, with NO_x_ ranging from <1–5 *μ*mol L^−1^, NO_2_ and NH_4_ almost always <0.5 *μ*mol L^−1^ and often close to or below detection limits, and PO_4_ frequently <0.2 *μ*mol L^−1^. Si(OH)_4_ in the Singapore Strait showed a seasonal pattern inverse to that of salinity. In contrast, the Johor Strait mostly had NO_x_ > 5 *μ*mol L^−1^ and often >10 *μ*mol L^−1^, with NO_2_ regularly contributing more than 50% of NO_x_. NH_4_ mostly ranged at Pasir Ris from 1 to 8 *μ*mol L^−1^, and at Sembawang mostly around 10–40 *μ*mol L^−1^, reaching as high as 75 *μ*mol L^−1^. PO_4_ in the Johor Strait was generally in the range of 0.5–3.0 *μ*mol L^−1^, but occasionally dropped to <0.1 *μ*mol L^−1^. Higher values were usually found during the SW monsoon. In the Singapore Strait, NO_x_ and PO_4_ were generally highest just before or during the SW monsoon, and lowest during the NE monsoon. The Johor Strait displayed less coherent seasonal patterns, with NO_x_ and PO_4_ showing opposite seasonal trends in Sembawang, and no clear pattern for Si(OH)_4_. There were no differences in total precipitation for the 7 days prior to sampling between the Johor and Singapore Straits throughout the year, with values ranging from 0 to 128 mm (Supplementary Data S1).

Prokaryotic abundance was different between the Singapore and Johor Straits (Supplementary Data S1, Fig. S2), with abundances ranging from 0.4 × 10^6^ to 1.6 × 10^6^ cells mL^−1^ and 1.1 × 10^6^ to 9.4 × 10^6^ cells mL^−1^, respectively. In general, higher prokaryotic counts were recorded at Sembawang.

Chlorophyll concentration varied substantially between sites, broadly reflecting the concentration of nutrients: low values were found in the Singapore Strait (mostly <2 *μ*g L^−1^, but up to 13 *μ*g L^−1^), while the Johor Strait rarely had less than 10 *μ*g L^−1^, and up to 94 *μ*g L^−1^ (Fig. 1, Supplementary Data S1). In the Singapore Strait, chlorophyll was lowest during the SW monsoon and highest during inter-monsoon 1 or the NE monsoon, while in the Johor Strait, concentrations were highest during the NE monsoon or inter-monsoon 2, and lower during the SW monsoon.

### Microbial community composition of Singapore coastal waters

We analyzed the microbial community present in the 0.2–150 *μ*m size fraction. A similar approach has been implemented in global studies such as the Ocean Sampling Day project^42^. A total of 70 samples, 30 from the Singapore Strait and 40 from the Johor Strait from February 2017 to July 2018 were analyzed by rRNA metabarcoding using primers that amplify both prokaryotic 16S and eukaryotic 18S rRNA gene^26^. After quality control filtering, end-pair joining and chimera filtering, a total of 5,036,783 sequences were retained with an average of 71,954 sequences per sample which were assigned to 2,571 ASVs, once Metazoa and plastid sequences were removed (Supplementary Data S2).

**Figure 2:**
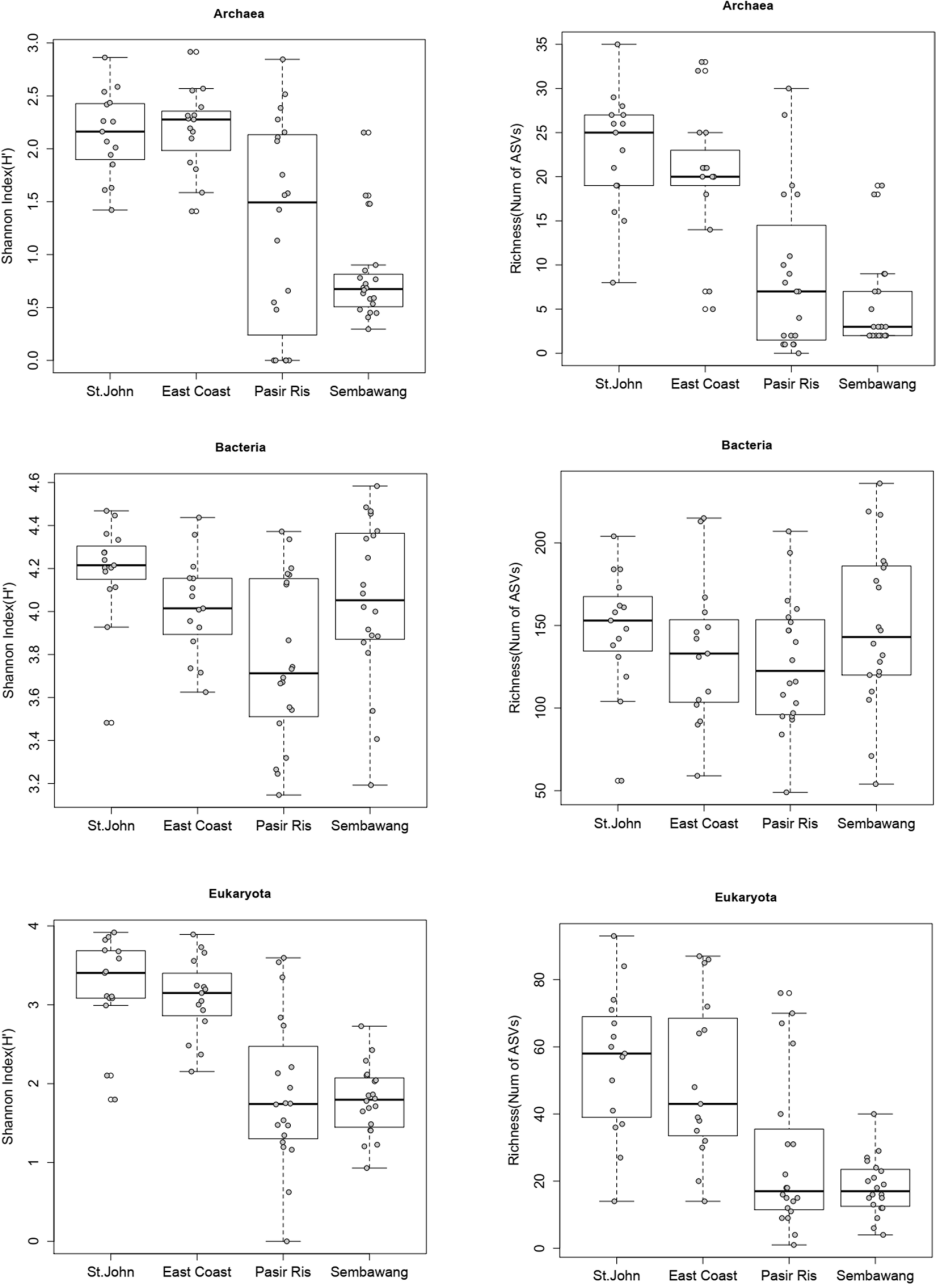
Shannon Index (H′, left) and Richness (Number of ASVs, right) of microbial communities at 4 stations in Singapore coastal waters for Archaea (top, **A**,**B**), Bacteria (middle, **C**,**D**) and Eukaryota (bottom, **E**,**F**).

Based on the Shannon index (H′), Singapore Strait communities generally had a higher diversity than those from the Johor Strait for Archaea and Eukaryota, but not for Bacteria (Fig. 2). The richness, inferred from the number of ASVs, was also generally higher in the Singapore Strait for Archaea and Eukaryota (Fig. 2).

**Figure 3:**
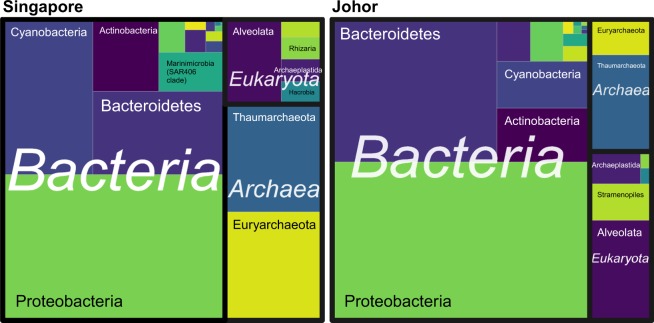
Tree map with the major taxonomic groups in Singapore and Johor Straits.

The taxonomic composition of Bacteria, Archaea and Eukaryota communities varied between straits (Figs 3, S3 and S4). At the domain level, Bacteria dominated both straits but Archaea were more prevalent than Eukaryota in the Singapore Strait compared to the Johor Strait (Fig. 3).

Among prokaryotes, Cyanobacteria, specific proteobacterial groups and the archaeal phyla Euryarchaeota and Thaumarchaeota were over-represented in the Singapore Strait. Within the Archaea, the phylum Thaumarchaeota was mainly represented by the order Nitrosupumilales, which was present in both the Singapore and Johor Straits, but had a higher relative proportion in the Singapore Strait (Fig. S4). In the Singapore Strait, both *Candidatus* Nitrosopelagicus and *Candidatus* Nitrosopumilus were abundant while *Candidatus* Nitrosopumilus was abundant in the Johor Strait (Fig. S4). Euryarchaeota were mostly found in the Singapore Strait, with many ASVs assigned to marine group II Euryarchaeota (MGII). Marine group III Euryarchaeota (MG-III) were also present in the Singapore Strait, but in lower proportion, and absent in most samples from the Johor Strait. Other prevalent members of the microbial communities of the Singapore Strait included the alphaproteobacterial group SAR11, the gammaproteobacterial group SAR86, the deltaproteobacterial group SAR324 and the candidate actinobacterial genus *Actinomarina*. In the Johor Strait, Proteobacteria and Bacteroidetes were the most abundant groups (Fig. S4). The most abundant family within the Bacteroidetes in Johor included the *Flavobacteriaceae* and the NS5 marine group. Within the Proteobacteria, there was a high abundance of reads assigned to *Roseobacter* strain HIMB11, to members of the gammaproteobacterial OM60/NOR5 clade, Cellvibrionales (in particular members of the genus *Luminiphilus*) and the family *Burkholderiaceae* (Figs S3, S4 and S6).

The eukaryotic planktonic community in both straits was dominated by Dinoflagellata (Alveolata), Ochrophyta (Stramenopiles, mostly diatoms) and Chlorophyta (Fig. S4C). The dominance of Alveolata is probably linked to the large number of rRNA copies they harbor compared to their cell size^43^. Among Dinoflagellata, Dinophyceae and Syndiniales were the main groups in the Singapore Strait, while the Johor Strait was dominated by Dinophyceae (Fig. S4C). Most of Dinophyceae reads from the Johor Strait were affiliated with the order Gymnodiniales (e.g. *Gyrodinium* and *Woloszynskia*) and Gonyaulacales, while the Singapore Strait reads were affiliated with uncultured dinoflagellate sequences (Fig. S5). For example, the sequence of the most abundant Dinophyceae ASV (0115) in the Singapore Strait is 100% similar to that of a marine eukaryote clone o10.5–16 (KX532243) from the South China Sea (Supplementary Data S2). The Dinophyceae community in the Singapore Strait was more diverse than in the Johor Strait, where two ASVs (0011, *Gyrodinium* and 0043, *Gonyaulax*) dominated the whole community (Fig. S5). These ASVs were also within the top 50 most abundant ASV when considering the whole dataset (Bacteria, Eukaryota and Archaea).

Reads assigned to the marine parasites Syndiniales were nearly absent in the Johor Strait. The most abundant Syndiniales ASV found in the Singapore strait were absent in Johor (Fig. S5 Supplementary Data S2). Syndiniales are divided into 5 main groups^44^. In the Singapore Strait, Syndiniales ASVs were mainly affiliated to the highly diverse group II (Fig. S7), which contains only one genus formally described, *Amoebophrya*, which infects a wide range of dinoflagellate hosts^44^. Most Syndiniales ASVs could only be assigned to uncultured clades known from environmental sequences (Supplementary Data S2). For example, the sequence of the main Syndiniales ASV (0285) from the Singapore Strait was 100% similar to the uncultured eukaryote clone ST5900.074 (KF130025) obtained from the South China Sea (Supplementary Data S2).

Among the other Alveolata groups, very few reads were assigned to Apicomplexa (e.g Gregarines and Perkinsea, Supplementary Data S2). Ciliophora ASVs were found in both Straits, but more abundant in the Singapore Strait (Fig. S4C) and mainly assigned to the class Spirotrichea, with the genera *Parastrombidinopsis* and *Pelagostrobilidium* present in the Johor Strait *vs. Strombidiida* and the *incertae sedis* genus *Mesodinium* in the Singapore Strait (Fig. S5).

In the Johor and the Singapore Straits, diatoms (Bacillariophyta, Ochrophyta) were the second and third most abundant group, respectively (Fig. S4). Mediophyceae (polar centrics diatoms) were well represented in the two straits while Coscinodiscophyceae (radial centrics diatoms) were mainly found in the Singapore Strait. *Cyclotella*, *Cerataulina* and *Thalassiosira* (all Mediophyceae) were the main genera found in the Johor Strait, while *Leptocylindrus* (Coscinodiscophyceae), *Guinardia* (Coscinodiscophyceae) and *Skeletonema* (Mediophyceae) dominated the Singapore Strait (Fig. S5). The highly diverse genus *Chaetoceros* (Mediophyceae) was the only dominant genus common to both straits. Only two ASVs with a low number of sequences were assigned to Bacillariophyceae (pennate diatoms), which contains genera such as the toxic blooming *Pseudo-nitzschia* (Supplementary Data S2). Other classes of Ochrophyta (except for ASV-2888 belonging to Raphidophyceae) were not detected based on nuclear 18S rRNA gene. However Pelagophycae, Dictyochophyceae and Chrysophyceae, all Ochrophyta classes, were detected based on plastid 16S rRNA gene (Supplementary Data S2). Stramenopile groups such as the diverse group of heterotrophic flagellates MAST (Marine stramenopiles) and fungus-like members of Oomycota and Labyrinthulea were also found in both straits, although in low abundance (Fig. S4C and Supplementary Data S2).

Mamiellophyceae and Trebouxiophyceae were the two main classes of Chlorophyta (green algae) found in the Singapore and Johor Straits, respectively. Trebouxiophyceae ASVs were assigned to the highly diversified marine and brackish water coccoid genus *Picochlorum* (Fig. S5), known for its broad halotolerance^45,46^. Members of the two widespread Mamiellophyceae genera, *Micromonas* and *Ostreococcus*, were found in both straits (Fig. S7 and Supplementary Data S2). *Micromonas* was the main photosynthetic genus in the Singapore Strait (Fig. S5) with two clades, one corresponding to the ubiquitous species *M. commoda*, and the other to the undescribed clade B5^47^, found at the two stations in the Singapore Strait all year around (Fig. S7). Two clades of *Ostreococcus* were found mainly at Pasir Ris station in the Johor Strait during the SW monsoon (Fig. S7): clade B, which seems to be the dominant clade in tropical waters^47^, and another clade that could be the new clade E, which had already been found in Singapore during the Ocean Sampling Day survey^47^ and for which no culture is available yet. The genus *Bathycoccus*, widespread in marine waters^47^, was less abundant than its Mamiellophyceae counterparts in Singapore waters, appearing only sporadically (Supplementary Data S2).

Within the supergroup Hacrobia, reads belonging to Haptophyta and Cryptophyta divisions were the most abundant. Among haptophytes, all ASVs belonged to the class Prymnesiophyceae (Fig. S5 and Supplementary Data S2) and were closely related to the widespread and non-calcifying genera *Chrysochromulina* and *Phaeocystis*, mostly in Singapore Strait. Only ASV-1427, with low abundance, was assigned to the order Isochrysidales that contains calcifying coccolithophorids (*Gephyrocapsa*) (Supplementary Data S2). Cryptophytes were mainly detected in the Singapore Strait and assigned to the genus *Geminigera* (Fig. S5).

Reads belonging to the supergroup Rhizaria were found in both straits. Radiolaria were dominant in the Singapore Strait but nearly absent in the Johor Strait. The most abundant Radiolaria ASV was assigned to the order Taxopodia clade RAD-B (Fig. S5). Rhizaria reads from the Johor Strait belonged to the division Cercozoa and were assigned to the Class Filosa-Imbricatea (Fig. S5)^48^.

### Factors influencing microbial community structure

**Figure 4:**
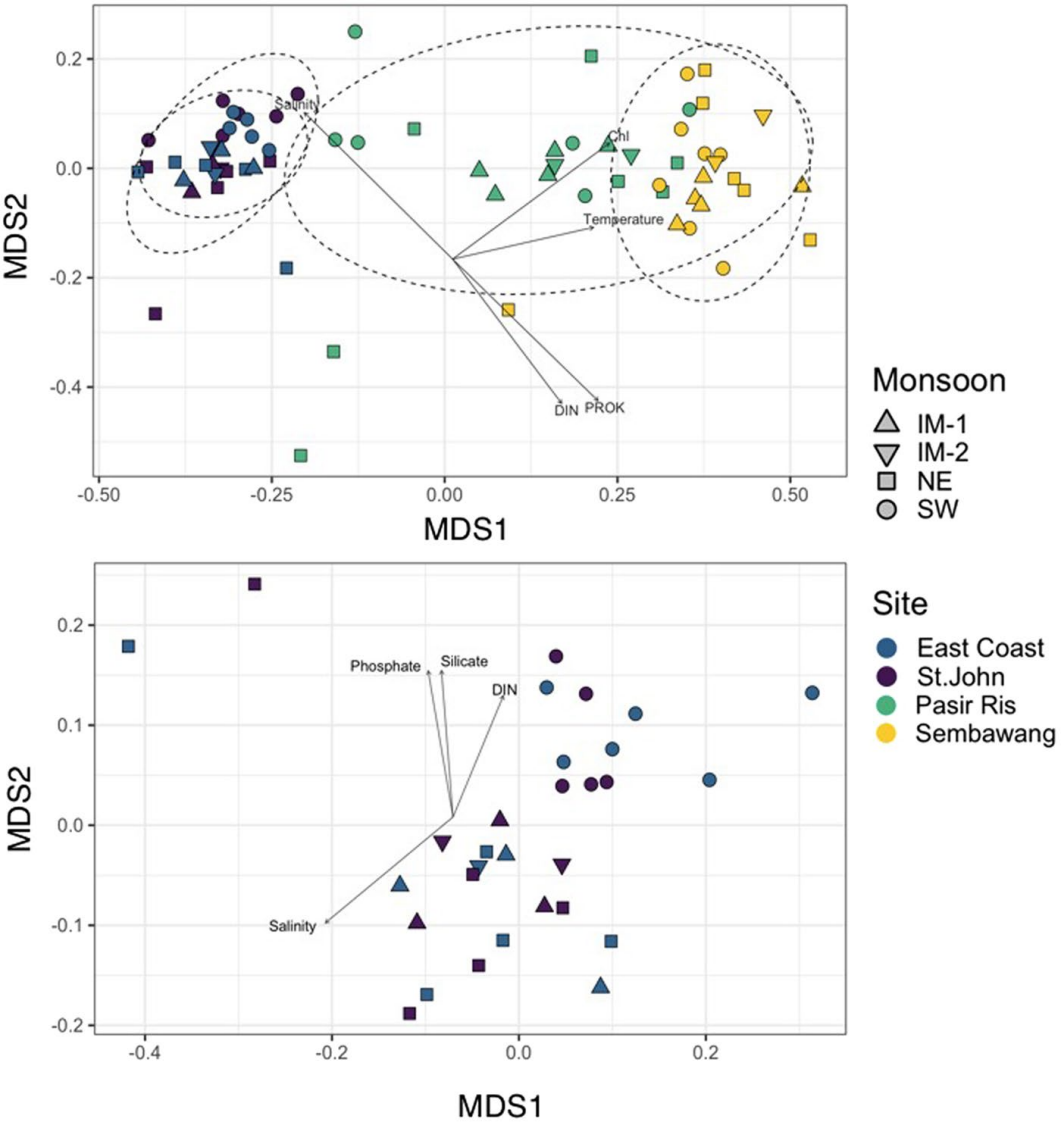
Non-metric multidimensional scaling (nMDS) analysis of Bray-Curtis similarity index. Each sample is labelled by location and monsoon period. Arrows represent environmental parameters with p < 0.001 when performing an *envfit* analysis The ellipses represent 95% confidence intervals for samples collected at the same station. Top. All stations. Bottom. Only Singapore Strait samples (STJ and EC stations).

Non-metric multidimensional scaling analysis (nMDS) of microbial community composition revealed that samples clustered based on strait (Fig. 4). While there was little dissimilarity between the St. John and East Coast sites within the Singapore Strait cluster, there was a clear separation between the Johor Strait sampling sites (Sembawang and Pasir Ris stations). An analysis of similarity (ANOSIM) based on different grouping factors (strait, site, monsoon) confirmed significant differences based on strait and site (Table 1). Envfit analysis revealed that most environmental factors (salinity, temperature, DIN, NH_4_) and biotic factors (chlorophyll *a* and prokaryotic abundance) were significantly correlated (p < 0.05) to the dissimilarity between Straits (Fig. 4).

**Table 1:** ANOSIM analysis.

Sample set	Grouping	R	Significance
All sites	Strait	0.802	0.001
	Site	0.652	0.001
	Monsoon	0.005	0.340
Singapore Strait	Site	−0.072	0.498
	Monsoon	0.270	0.002
Johor Strait	Site	0.421	0.001
	Monsoon	−0.026	0.675

An nMDS analysis taking only into account the Singapore Strait displayed little overlap between the SW monsoon and the others monsoon periods (Fig. 4). ANOSIM confirmed that there was a significant difference based on monsoon (Table 1). PO_4_, DIN and Si(OH)_4_ were the most significant environmental variables influencing separation of SW-Monsoon cluster from other monsoon periods (p < 0.1, Fig. 4). In contrast, the nMDS restricted to Johor Strait samples did not show clustering based on monsoon (Fig. S8) and ANOSIM demonstrated a significant difference only based on site (Table 1).

### Temporal variability in Singapore coastal water communities

Seasonal variability associated with monsoon was observed in the Singapore Strait. Linear regression models implemented in Gneiss analysis indicated a significant difference between communities in the SW and NE monsoon regime (Figure 4-top), with the most significant change being an increase in the relative abundance of members of the order Nitrosopumilaceae during the SW monsoon (Fig. S6).

A more detailed differential analysis using DESeq2^38^ revealed that the abundance of 71 specific taxa was influenced by the NE or SW monsoon. Among those ASVs related to Alteromonadales (0292, 0512, 0747), Pirellulales (0514, 0490), SAR11 (0217, 0407, 0448, 0130, 0181, 0280, 0074, 0054), SAR116 (0371) and *Synechococcus* clade CRD1 (0277) were associated with the SW monsoon, while the ASVs related to Desulforculaceae (0772, 0921), Opitutales MB11C04 marine group (0819, 0274) and *Cyanobium* (0424, 0470) were associated with the NE monsoon. For the archaea, different ASVs assigned to the Marine Group II and Marine Group III showed a change in their abundance by monsoon. ASVs related to Marine Group II (0398, 0185, 0358, 0101) and Marine Group III (0297) were more abundant during the SW monsoon while ASVs related to Marine Group II (0403,441) and Marine Group III (0423) were more abundant during the NE monsoon. An abundant archaeal ASV (0005) assigned to *Candidatus* Nitrosopumilus also showed an increase during the SW monsoon. Among eukaryotes, thirteen ASVs had their abundance influenced by the monsoon according to DESeq2 analysis (Fig. S9). ASVs assigned to Syndiniales group I (clades 1 and 2) and group II (clades 7 and 10-11), The abundance of Mediophyceae and the ciliate genus *Mesodinium* increased during the SW monsoon (Fig. S9). During the NE monsoon an increase in abundance of ASVs related to the Syndiniales group I (clades 1 and 4) and II (clade 16), the dinoflagellate order Gymnodiniales, the diatom genus *Guinardia* and the ciliate Class Spirotrichea was observed. Within these 13 ASVs, only 3 (0532, 0380 and 0324) were among the top 30 most abundant ASVs in the Singapore Strait (Fig. S5).

When compared to Singapore Strait, the Johor Strait had larger monthly variations in community structure which did not correlate with the Monsoon season. The volatility plot of the first-distances analysis of the unweighted Unifrac matrix (Fig. S10), which can be interpreted as the month-to-month change in the composition of microbial communities, were generally higher for samples from the Johor Strait (Sembawang and Pasir Ris) and lower for the Singapore Strait sites (St. John and East Coast). The comparison of Figs 1 and S10 revealed that, in the Johor Strait, peaks in volatility corresponded to large month-to-month variations in salinity.

## Discussion

### Johor Strait microbial community is influenced by short term events

The water in Johor Strait was generally more brackish compared to the Singapore Strait and did not display a clearly defined seasonal pattern in salinity (Fig. 1). The lower salinity at Sembawang compared to Pasir Ris also suggests that the central portion of the Johor Strait is affected by land-runoff from storm drains, reservoirs and smaller rivers such as the Sungai Tebrau, located just north of Sembawang station. The oscillation in salinity was also reflected in the eukaryotic community composition, which harbored brackish water (e.g *Cyclotella*) and marine euryhaline groups (e.g *Picochlorum*). Groups known to have an exclusive marine lifestyle such as Syndiniales and *Micromonas* were absent in Sembawang samples and only sporadically found in samples from Pasir Ris (Fig. S7). On the other hand, the cosmopolitan genus *Gyrodinium*, known to inhabit marine, brackish and freshwaters, was found at both Johor stations (Fig. S7).

The Johor Strait is clearly eutrophic compared to the Singapore Strait, with consistently higher nutrient and chlorophyll concentrations. Higher concentrations of NO_2_ and NH_3_, and the higher cells counts, indicate that heterotrophic recycling is likely more pronounced in the Johor Strait, consistent with eutrophication. It is unclear to what extent the nutrient concentrations in the Johor Strait are controlled by direct inputs *via* run-off, or whether sedimentary recycling processes are significant. The fact that the concentration of DIN is inversely correlated to salinity in the Johor Strait (Fig. S11) but not to precipitation (data not shown) suggests that reservoir run-off processes may play an important role. Moreover, compared to the Singapore Strait, the Johor Strait exhibited a higher abundance of copiotrophic families of microbes such as the *Flavobactericeae*, the *Burkholderiaceae*, taxa affiliated with the *Roseobacter* and the OM60/NOR5 clades. All these groups have been previously implicated in nutrient remineralization and rapid responses to pulses of organic carbon such as those resulting from phytoplankton blooms and coastal runoff^49,50^. The most abundant prokaryotic ASV in the Johor Strait *Roseobacter* strain HIMB11 (Fig. S5) was previously shown to have the genomic potential for degradation of algal-derived compound such as DMSP^51^. The *Burkholderiaceae* have also been shown to be one of the dominant groups in the network of urban waterways of Singapore^52^ which might represent a reservoir and source for their presence in Johor Strait.

A lower alpha diversity (Fig. 2) and a larger beta diversity (as shown in the nMDS plot, Fig. 4) was observed in the Johor compared to the Singapore Strait. The overall lower alpha diversity in the Johor Strait was caused by a decreased richness of Eukaryota and Archaea but not of Bacteria. The frequent and short pulse disturbances^53,54^ (short-term events with release of nutrients and sediments) experienced by the Johor Strait planktonic community seemed to affect its stability and ultimately the diversity of the system^55^. The clear separation between the Johor and Singapore Strait clusters in the nMDS plots suggests that limited exchange exists between the planktonic communities from the Johor and the Singapore straits as well as within the Johor Strait, as indicated by the cluster separation between Pasir Ris and Sembawang samples (Fig. 4). In the absence of mixing, it is possible that local effects might contribute to the development of locally adapted, low diversity communities. This hypothesis is further supported by observing that seasonal change in regional seawater circulation due to monsoon wind reversal did not affect the microbial community composition of the Johor Strait directly. The only seasonal trend we observed in the community, linked to the SW monsoon, was the presence of the two pico-phytoplanktonic genera *Ostreococcus* and *Micromonas* (clade B5) in Pasir Ris samples during July of 2017 and 2018 (Fig. S7).

### Singapore Strait community and seasonality

The Singapore Strait samples contained a higher proportion of autotrophic and oligotrophic microbes such as the Synechococcales, Nitrosupumilales, marine group II Euryarchaeota (MGII) and SAR11. The most prevalent cyanobacteria found in the Singapore Strait belonged to the order Synechococcales and were most closely related to *Synechococcus* representative of clade II, which has been identified as the dominant type in warm and oligotrophic waters^56^. The phylum Euryarchaeota found in high abundance in the Singapore Strait is related to Marine Group II, which is widely distributed in the oceanic euphotic zone^57,58^ and is the dominant planktonic Archaeon at the surface water of the South China Sea^59^. Marine group III Euryarchaeota (MG-III) was also present in the Singapore Strait, but in a lower proportion. This group is known to be prevalent in deep-sea waters, but few studies have reported their presence in the photic zone^60–62^. Other prevalent members of the microbial communities of the Singapore Strait included the Alphaproteobacteria clade SAR11, which has previously been found to dominate heterotrophic bacterial communities in coastal and open ocean environments^63–65^.

The microbial community shift in the Singapore Strait is synchronous with the seasonal reversal of ocean currents between the Java Sea and the South China Sea^16^ suggesting that the advection of microbial communities from different water basins might be causing this shift. During the SW monsoon, currents flow northward from the Java Sea^16^, bringing less saline water into the Singapore Strait due to the high precipitation over Sumatra, Borneo, and Java. The high freshwater input from these islands into the Java Sea is also the likely source of nutrients transported to Singapore at the start of the SW monsoon period (Fig. 1). The net northward water transport during the SW monsoon brings a community with a higher proportion of Archaea to the Singapore Strait, especially the ammonia-oxidizing archaea *Candidatus* Nitrosopelagicus and *Candidatus* Nitrosopumilus, presumably reflecting higher nutrient concentrations and greater dominance of heterotrophic recycling of organic matter. During the NE monsoon, the current direction reverses and water from the South China Sea flows into the Singapore Strait^16^, carrying more saline and more oligotrophic water with less terrestrial input into the Singapore Strait. Consequently, all nutrients decrease in concentration except for a small peak around the beginning of the NE monsoon (Fig. 1) that is probably driven by local run-off (since this is typically the period with the highest rainfall in Singapore).

While our study showed an increasing trend in nutrient concentration during the SW monsoon, the chlorophyll did not show any trend. Other parts of South East Asia have been shown to experience seasonal dynamics in chlorophyll concentration as a result of monsoon systems^11,13,14^. In contrast, the analysis of the ASVs that differ most significantly between the two monsoons suggested that the seasonal cycle does not affect the autotrophic prokaryotes but that ecotype replacement in the heterotrophic bacterial community. This replacement involves ASVs belonging to SAR11 clades, SAR406 and the NS4 and NS5 groups, which have been shown to display seasonal dynamics in temperate waters^66–70^.

Although few eukaryotic ASVs had their abundance increased or were present during either the SW or NE monsoon period, no clear seasonal pattern was observed among eukaryotes. *Micromonas*, the main photosynthetic picoeukaryotes in our dataset, have been reported as the dominant group in the sub-tropical waters of the South China Sea^71^ and off Taiwan^72^. In Taiwan, high abundance of clade B5 was detected during summer and autumn, with a peak in July when high temperature and irradiance, as well as oligotrophic conditions prevail^72^. In our study, *Micromonas* sp. clade B5 seems to have higher abundance during the NE and inter monsoon months (Fig. S7), a period when the water of the Singapore Strait tends to be more oligotrophic.

Members of the *Mesodinium* species complex are known to form periodic or recurrent non-toxic blooms (red tides)^73,74^. These ciliates can photosynthesize by acquiring and maintaining organelles from cryptophyte prey^75,76^. Cryptophytes are an important component of phytoplankton communities in coastal ecosystems, especially estuaries environments^77,78^. High abundances of cryptophytes have been associated with either preceding^79^ or co-occurring peaks^80^ of *Mesodinium* in coastal ecosystems. Although *Mesodinium* and the cryptophyte species complex *Geminigera* were among the abundant groups in Singapore strait, we did not observe *Geminigera* - *Mesodinium* dynamics in our dataset. Also, to our knowledge, red tides have not been reported in the Singapore Strait.

Blooms of *Coscinosdiscus* and *Chaetoceros*, during the NE and inter-monsoon periods, were reported in the Singapore Strait 70 years ago^18^. Although these taxa were among the dominant phytoplankton group that we found in the strait, neither a seasonal trend nor a bloom were observed during the present study. During the 1968 SW monsoon, a bloom of pennate diatoms (Bacillariophyceae) was also reported^18^, and this group is known to be part of the phytoplankton community in the Singapore Strait^17^. Surprisingly, very few reads in our dataset were assigned to Bacillariophyceae, probably due to the fact that the forward primer used displays one mismatch to all Ochrophyta (data not shown). This is confirmed by the fact that this group was detected through its plastid 16S rRNA gene sequences (Supplementary Data S2). As this is the first long-term study of the protist communities in the waters of Singapore since the early 50’s^18^, it is impossible to known whether either the absence of blooms today or the appearance of blooms in the past are anomalies of the ecosystem.

## Conclusion

In this 18-month long study we successfully captured the environmental characteristics of two Straits with different trophic status. In the eutrophic Johor Strait, large environmental fluctuations were observed throughout the year, yet no recurring seasonal pattern could be detected in the microbial community composition. Conversely, the Singapore Strait showed a seasonal variability which might be a result of the different monsoon regimes. Our study suggests that even in the vicinity of the Equator, where irradiance and temperature show little variation, seasonal trends are reflected in the microbial community composition in relation to monsoon alternation. Based on these findings, we argue that longer sustained observations over a broader spatial scale are needed, in equatorial waters, to identify the patterns of microbial community assembly and the long term trajectories in the seasonal succession of specific taxonomic groups of Bacteria, Archaea and Eukaryota.

## Supplementary information

Supplementary information.

## Data Availability

Raw sequencing data have been deposited to GenBank under Bioproject number PRJNA497851. Processed data and scripts are available from https://github.com/slimelab/Singapore-metabarcodes.
