# Supplementary material for: Temporal and spatial dynamics of Bacteria, Archaea and protists in equatorial coastal waters
Source: Sci Rep. 2019 Nov 8;9:16390. doi: 10.1038/s41598-019-52648-x (PMC6841670; doi:10.1038/s41598-019-52648-x)
Supplement: Supplementary file 1 — Supplementary information. [file 41598_2019_52648_MOESM1_ESM.pdf]

Temporal and spatial dynamics of Bacteria, Archaea and protists in equatorial coastal waters

Caroline Chénard[1], Winona Wijaya[1], Daniel Vaulot[2, 1], Adriana Lopes dos Santos[1, 3], Patrick Martin[1], Avneet Kaur[1], and Federico M Lauro[1, 4 *]

[1]Asian School of the Environment, Nanyang Technological University, 50 Nanyang Avenue, Singapore 639798

[2]Sorbonne Université, CNRS, UMR7144, Ecology of Marine Plankton team, Station Biologique de Roscoff, 29680 Roscoff, France

[3]GEMA Center for Genomics, Ecology & Environment, Universidad Mayor, Camino La Pirámide, 5750, Huechuraba, Santiago, Chile

[4]Singapore Centre for Environmental Life Sciences Engineering (SCELSE), Nanyang Technological University, 60 Nanyang Dr, Singapore 637551

[*]Corresponding author: Federico M. Lauro, 50 Nanyang Avenue, Singapore 639798, Tel: (65) 6790-9845, Fax: (65) 6515-6751, Email: flauro@ntu.edu.sg

Supplementary material

All supplementary material is available at https://github.com/slimelab/Singapore-metabarcodes

Supplementary Data

Supplementary Data S1: (R/Singapore metadata.xlsx) List of samples collected with environmental parameters.

Supplementary Data S2 (R/Singapore ASV_table.xlsx). Sheet ASV: List of ASVs with taxonomic affiliation, assignment bootstrap values for eukaryotes, sequence and number of reads in each sample (only samples from four stations,STJ, EC, SBW and PR are used in this paper). Sheet Blast eukaryotes: Summary of BLAST assignments against PR^2 and GenBank for Eukaryota ASVs (nuclear 18S rRNA).

Supplementary Figures

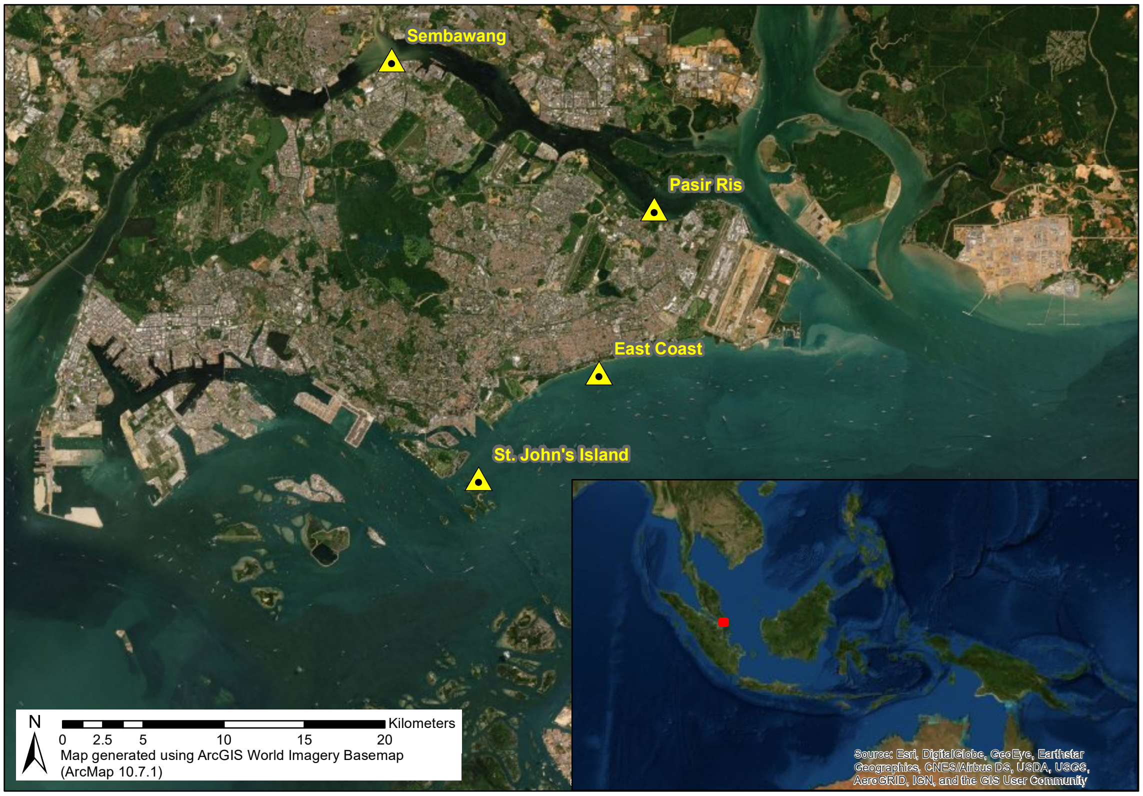

Figure S1. Map of Singapore showing the four stations sampled in this study. The map was generated with ArcMap version 10.7.1 using the following Esri ArcGIS Basemap: Esri. "World Imagery" 1:250,000. "Sampling Sites". Aug 31, 2019. https://services.arcgisonline.com/ArcGIS/rest/services/World_Imagery/MapServer

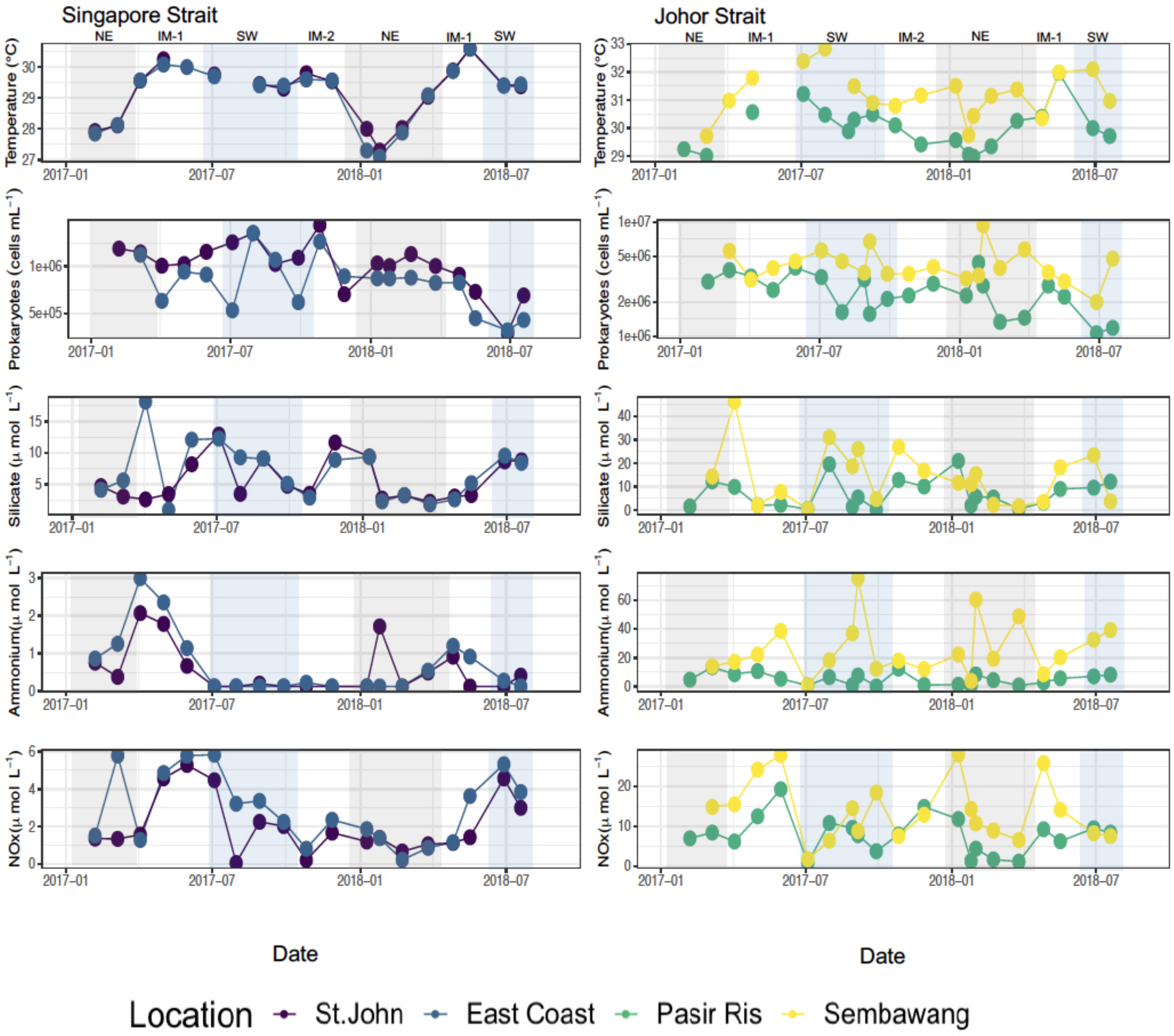

Figure S2. Temperature, prokaryotic abundance, silicates, ammonium and NO_x during the 18-month time series in Singapore coastal waters. Highlights in grey and blue represent NE period and SW periods respectively.

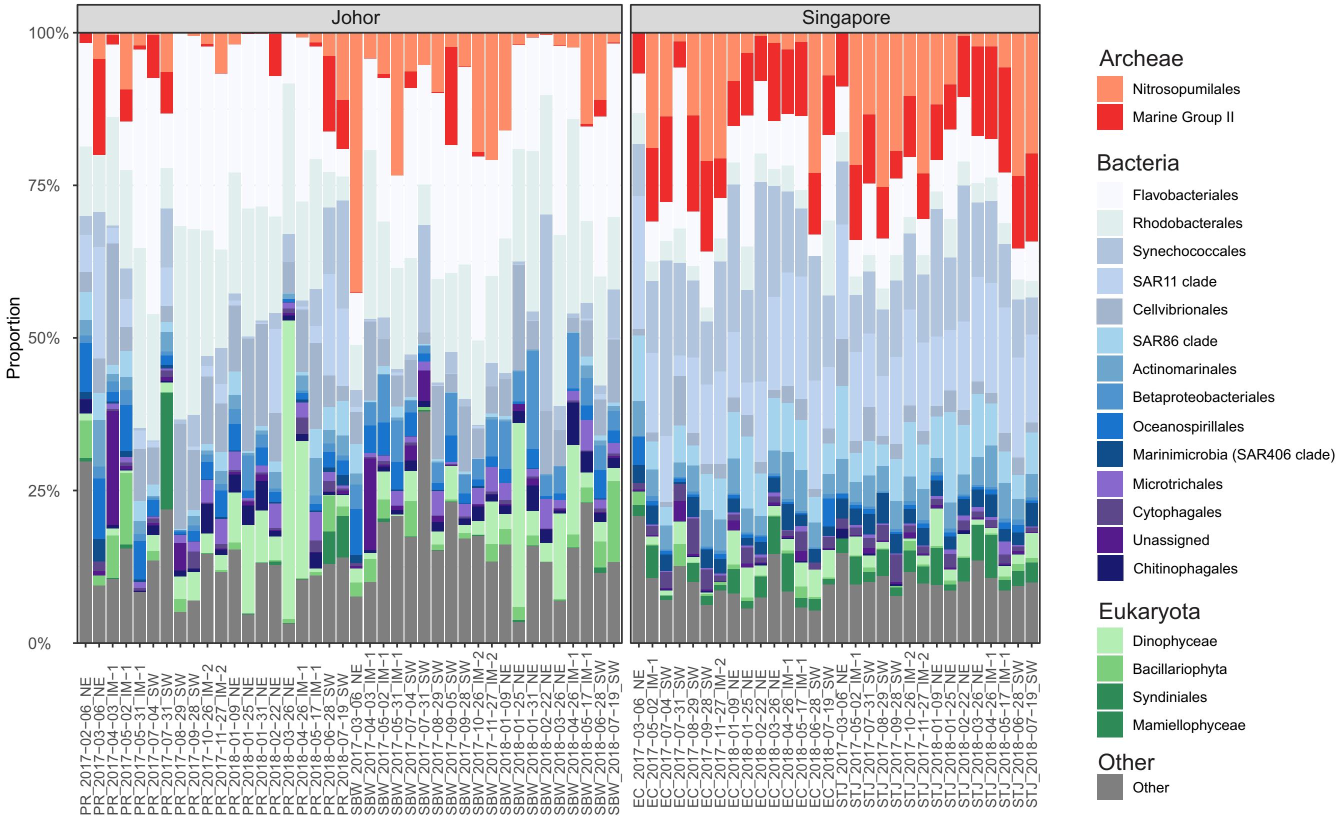

Figure S3. Taxonomic composition of the microbial communities in Singapore coastal waters focusing on the top 20 most abundant groups (Class level) for the three domains (Archaea, Bacteria, Eukaryota as shades of red, blue and green, respectively).

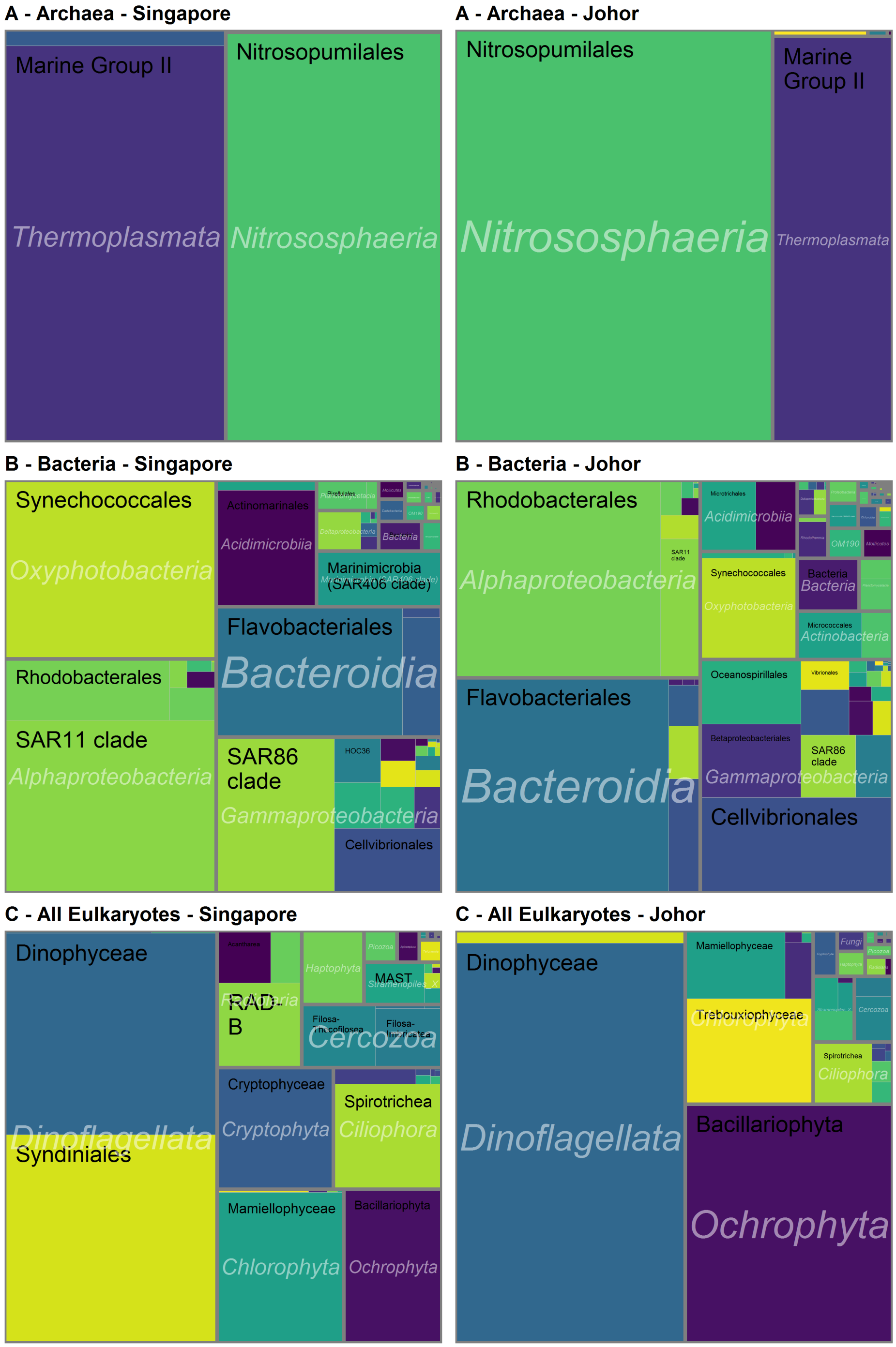

Figure S4. Tree map with the major taxonomic groups in Singapore and Johor Straits for the 3 domains: Archaea, Bacteria and Eukaryota.

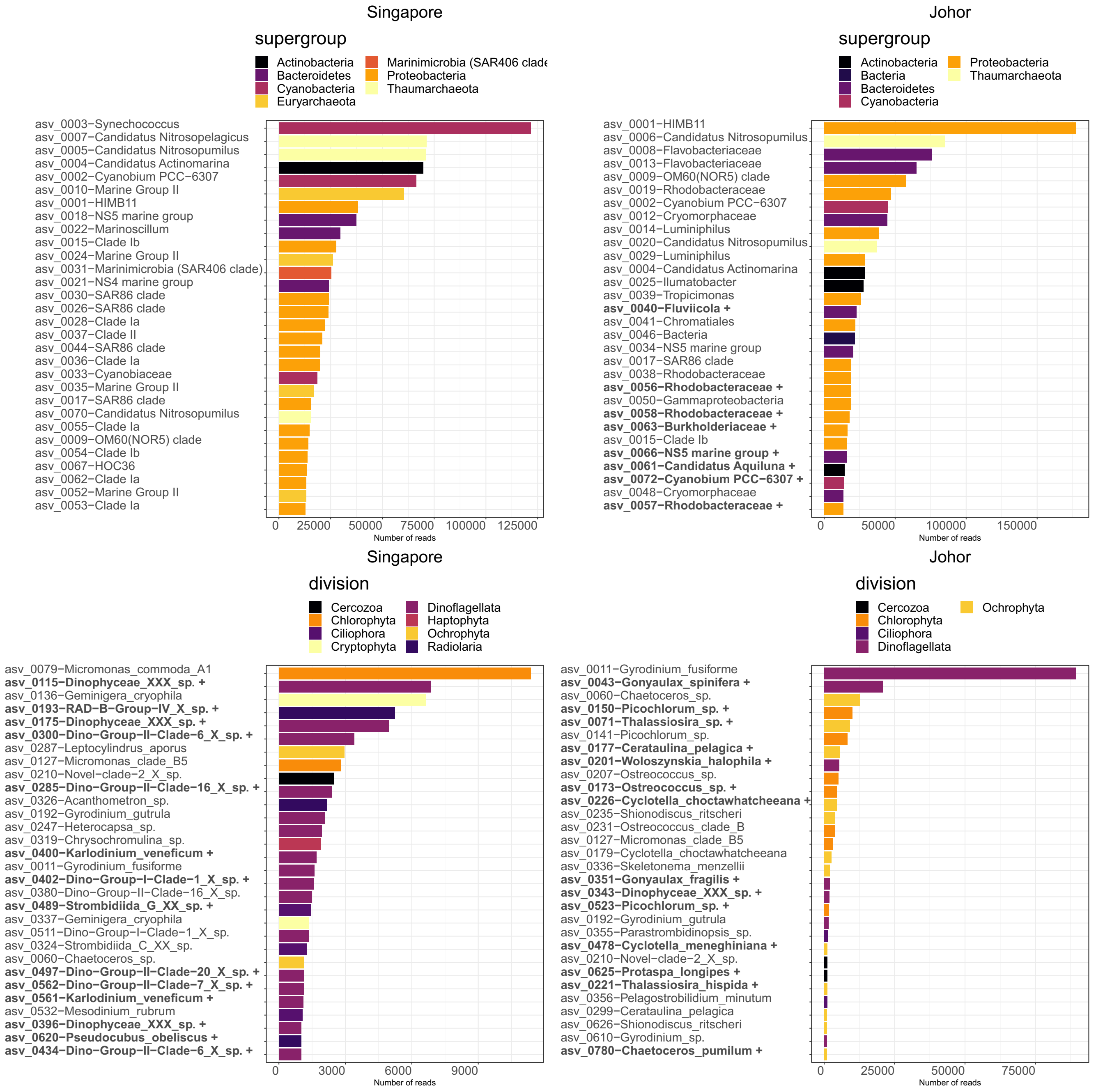

Figure S5. Thirty most abundant Archaea and Bacteria (top) and Eukaryota (bottom) ASVs for Singapore (left) and Johor (right) straits. ASVs that are unique to one Strait are in bold and labelled with a plus sign.

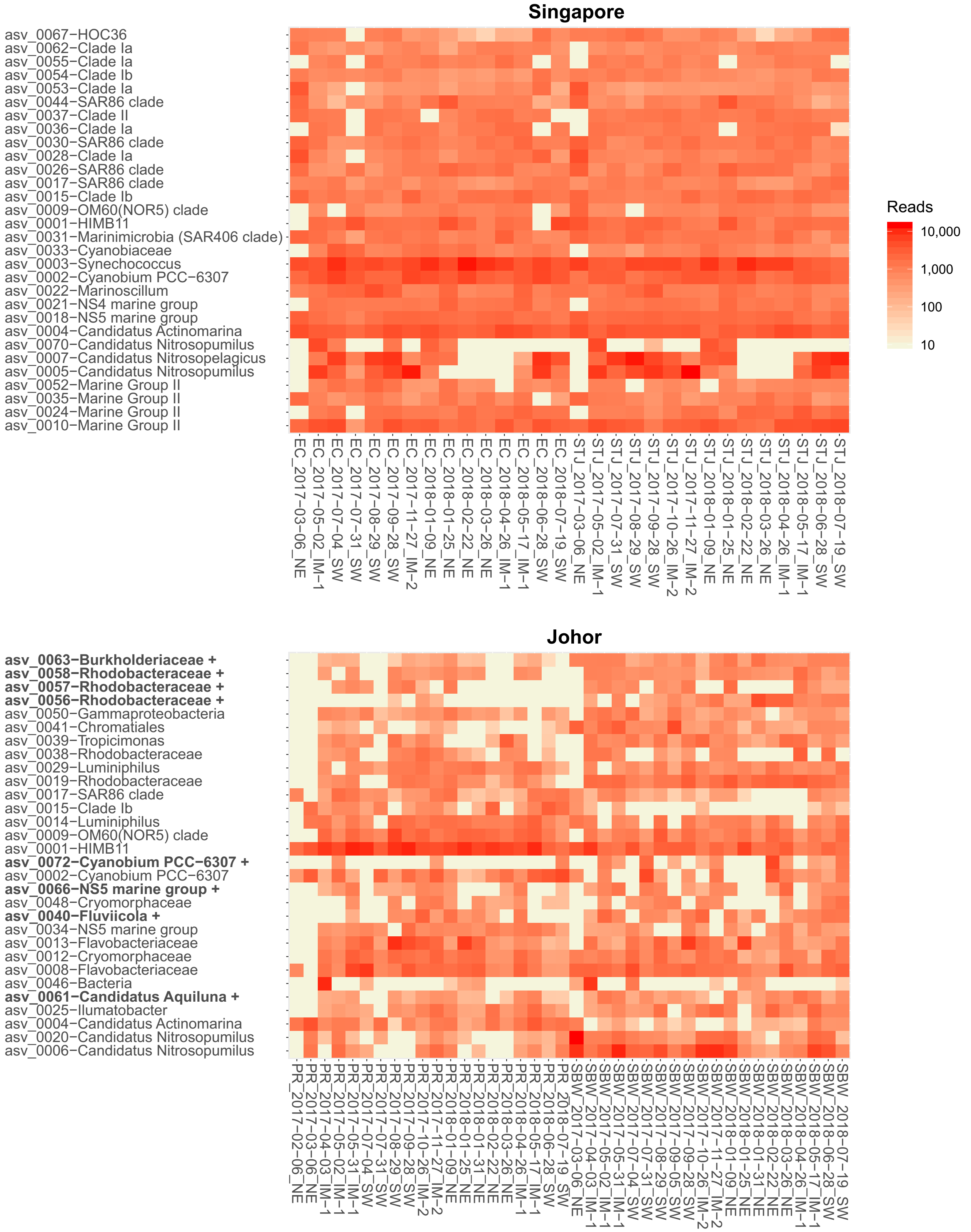

Figure S6. Sample heatmap of the thirty most abundant Archaea and Bacteria ASVs for Singapore (top) and Johor (bottom) straits. ASVs that represent more than 10 sequences and are unique to one Strait are in bold and labelled with a plus sign.

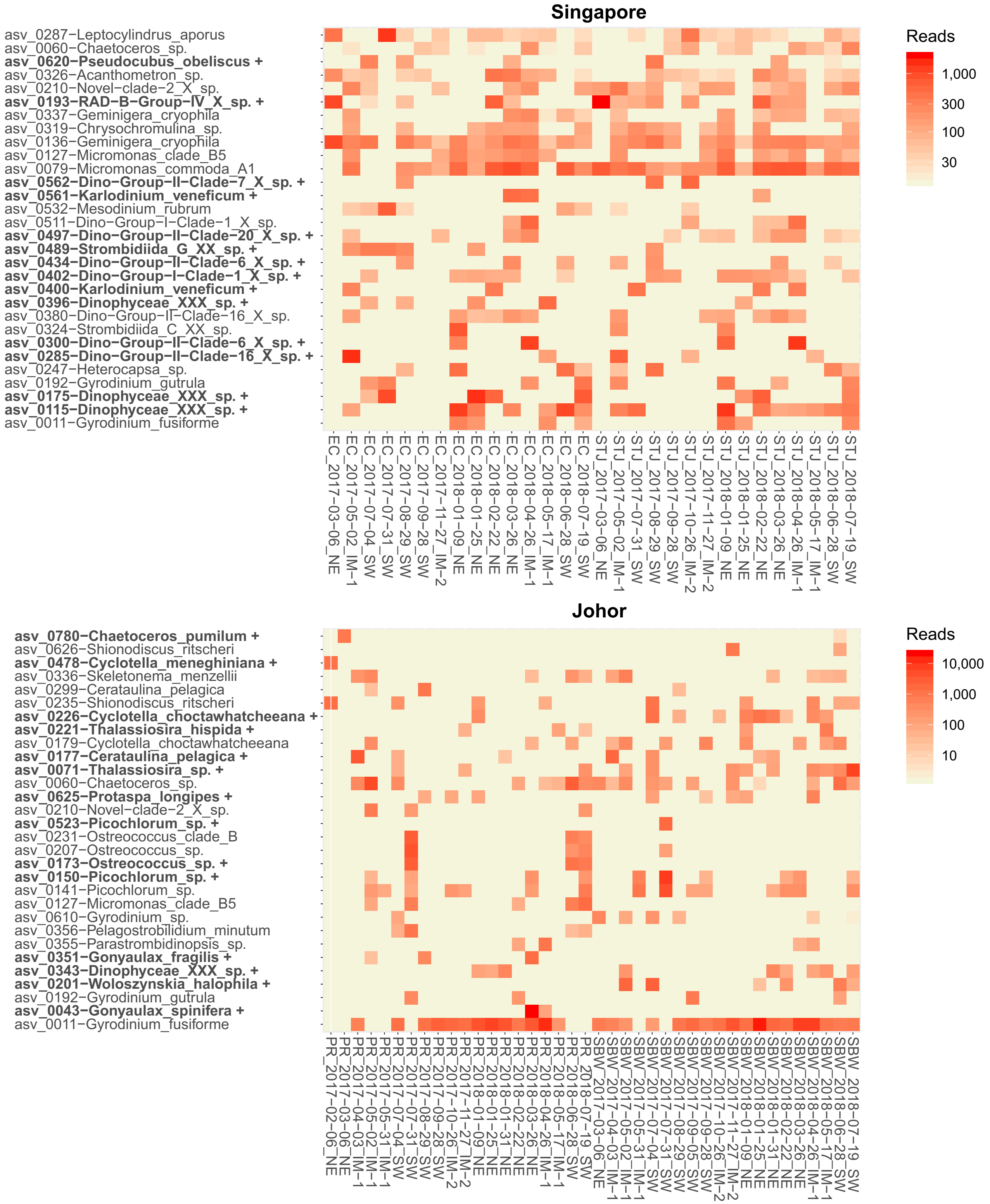

Figure S7. Sample heatmap of the thirty most abundant Eukaryota ASVs for Singapore (top) and Johor (bottom) straits. ASVs that are unique to one Strait are in bold and labelled with a plus sign.

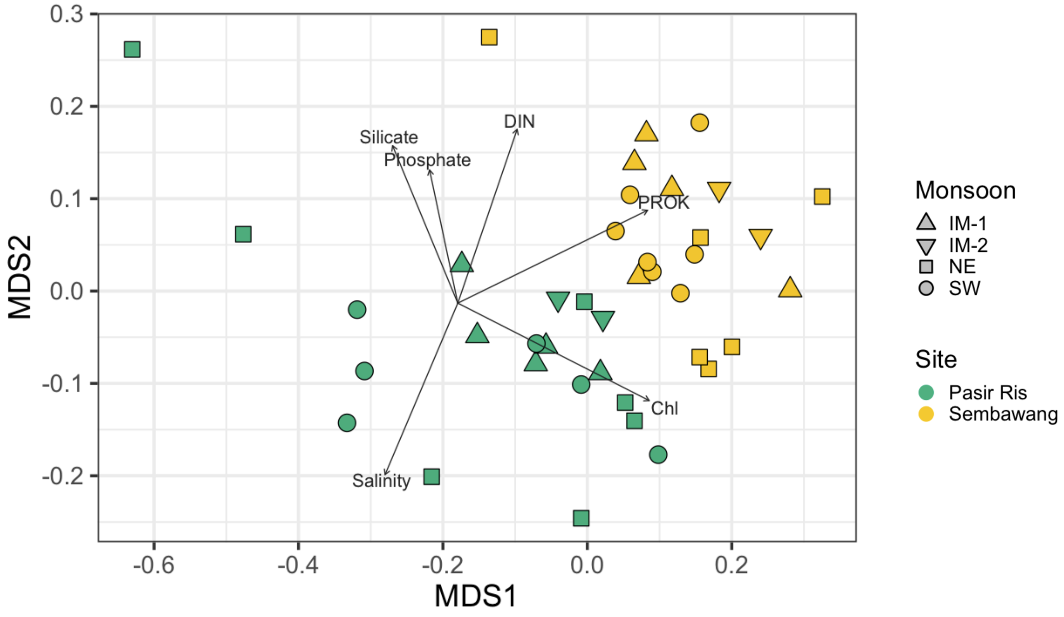

Figure S8. Non-metric multidimensional scaling (nMDS) of Bray-Curtis similarity index for Johor Strait (PR and SBW stations). Each sample is labelled based on location and monsoon period. The arrows represent environmental parameters with $p < 0.05$ when performing an *envfit* analysis.

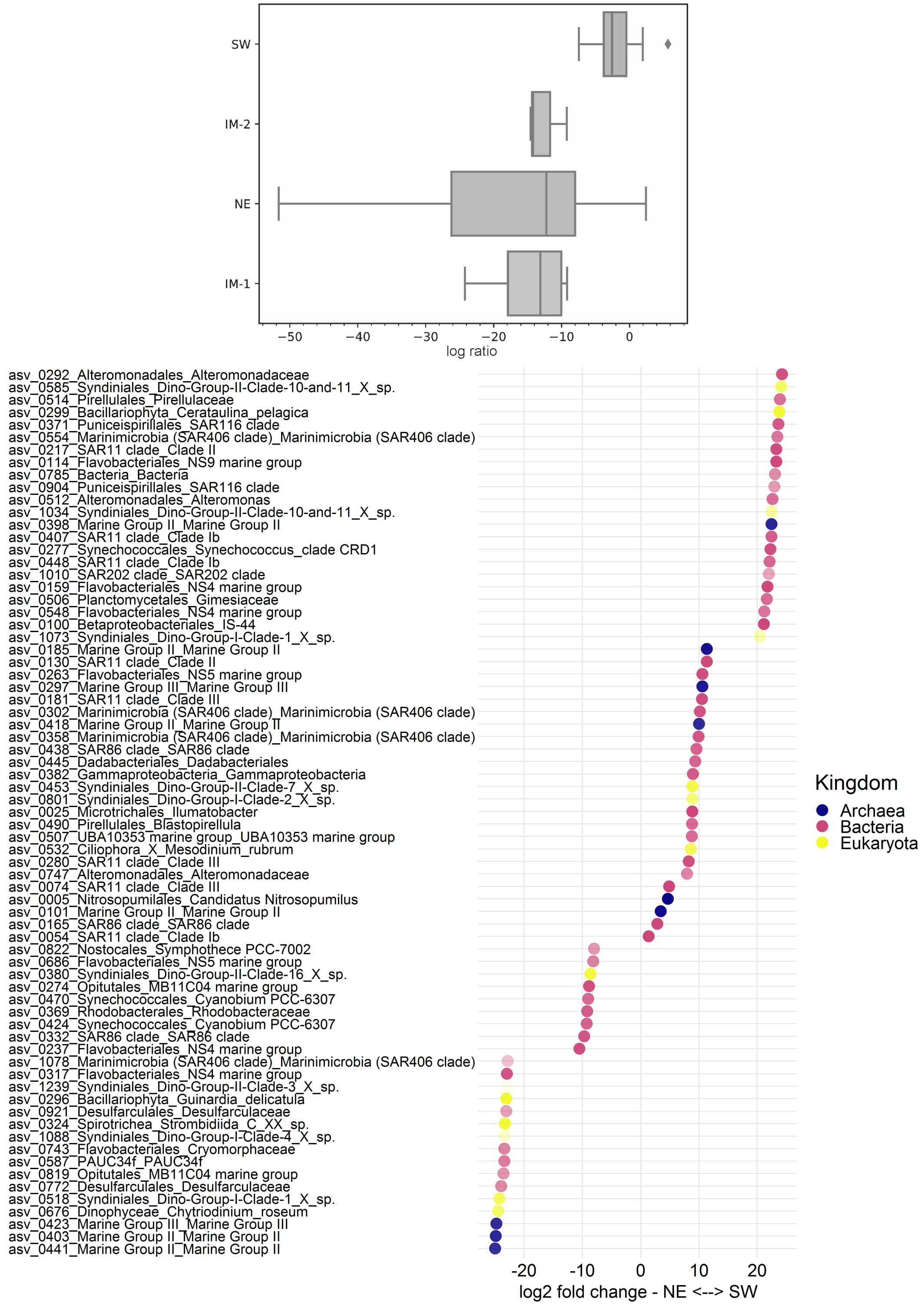

Figure S9. Top. Gneiss analysis for Singapore strait based on monsoon period. The Gneiss analysis uses balance trees to explain how different environmental variables affect the relative taxonomic distribution in a sample grouping. The log ratio of the global balances shown here tests whether the species distribution in different Monsoon seasons is significantly different and shows that the SW Monsoon communities are significantly different from the those of the NW and the inter-monsoon seasons. Bottom. Members of the communities that drive the difference between the NE and SW monsoons for Singapore Strait stations (21 samples) based on DESeq2 analysis using a threshold p-value < 0.01. Symbol transparency is inversely proportional to ASV rank.

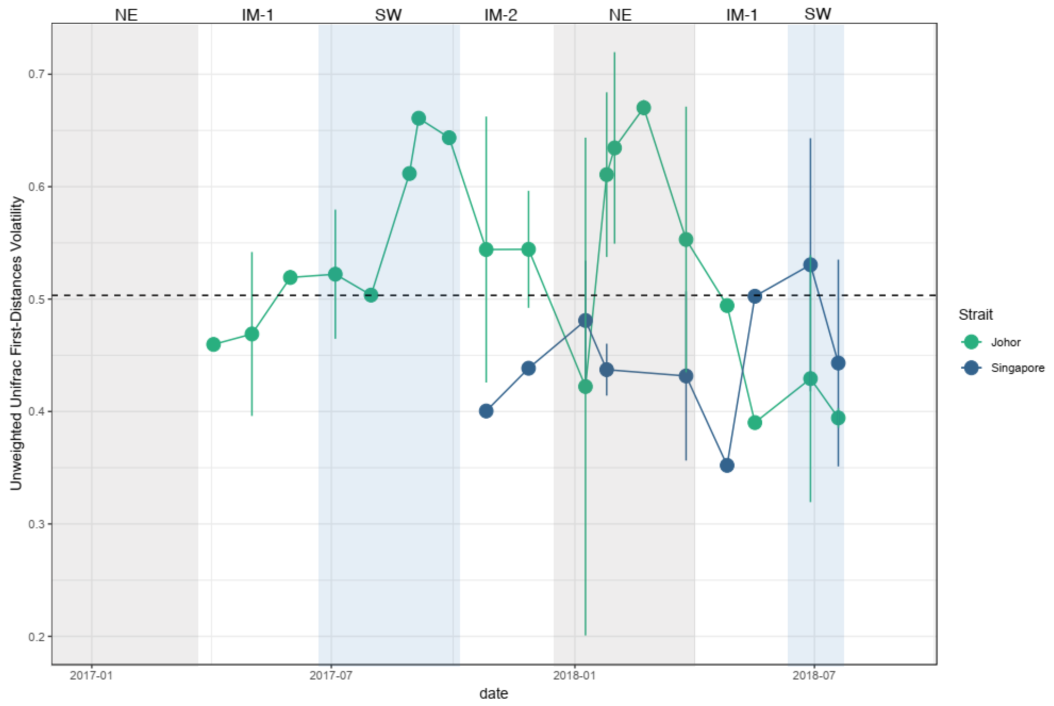

Figure S10. First-distances volatility values computed on the unweighted Unifrac distance matrix of the two Straits. The dotted line indicates the global average and error bars the individual sample dispersion from the Strait average

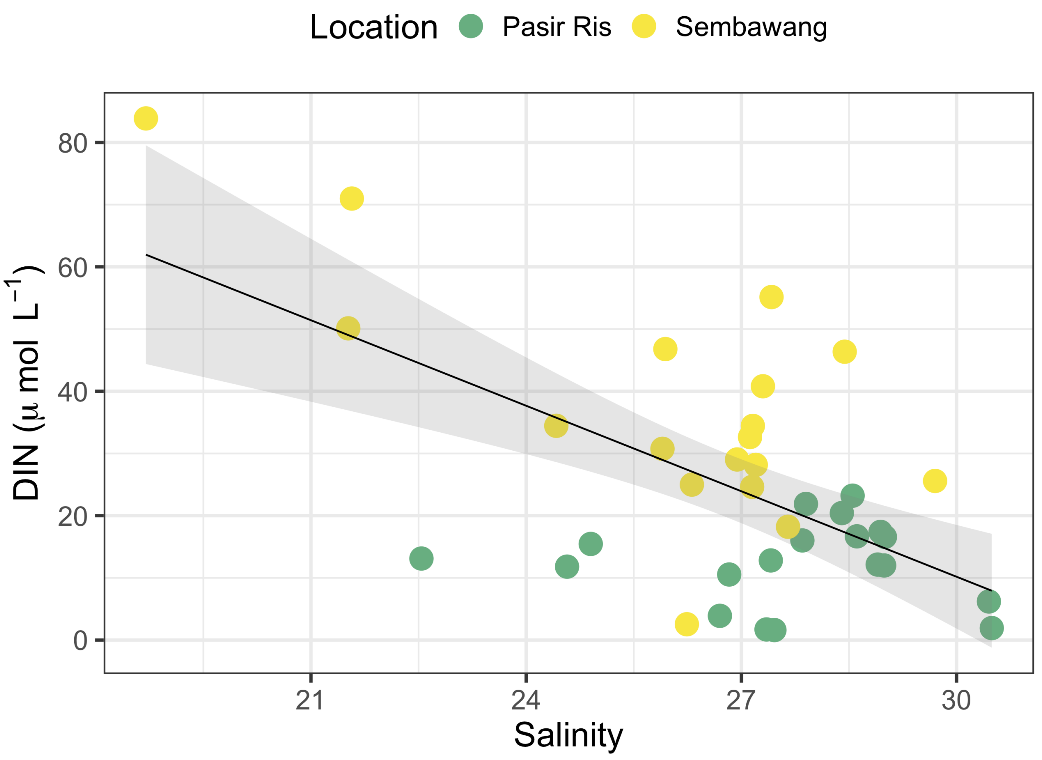

Figure S11. Correlation between DIN and salinity in Johor strait.
